# External Jugular Vein Slow-Flow Venous Malformation Presenting as a Swelling Over the Neck: A Report of a Rare Case

**DOI:** 10.7759/cureus.58973

**Published:** 2024-04-25

**Authors:** Sudhir Jayakar, Kondapalli Sri Sai Teja Sampath, Virendra Athavale, Siddharth Tiwari, Vinay Badangi

**Affiliations:** 1 General Surgery, Dr. D. Y. Patil Medical College, Hospital & Research Centre, Dr. D. Y. Patil Vidyapeeth, Pune, IND

**Keywords:** arteriovenous malformations, rare occurrence, external jugular vein malformation, neck trauma, external jugular vein aneurysm, head neck surgery, rare vascular malformations, rare head and neck, venous malformations

## Abstract

Vascular malformations originating from the wall of the external jugular vein are exceedingly uncommon. We present a unique case of a venous malformation arising from the external jugular vein, successfully treated through surgical excision with no subsequent recurrence. This case highlights the importance of early diagnosis and timely intervention in managing such rare clinical entities without any resulting morbidity.

## Introduction

In 1982, Mulliken and Glowacki proposed a classification of vascular lesions based on endothelial characteristics [1] in which vascular lesions were classified into two major groups: infantile hemangiomas and vascular malformations. Hemangiomas are characterized by endothelial cellular hyperplasia and proliferation, characteristically grow rapidly for six to eight months after birth, and then regress to variable extent [2]. On the other hand, vascular malformations have a normal endothelial cell cycle and do not involute [3]. Venous malformations (VMs) are rare abnormalities that may manifest as isolated neck masses in adults. Among the uncommon cases are cervicofacial VMs originating from the external jugular vein, with only a few instances documented in the medical literature [4]. In such cases, patients often seek treatment due to cosmetic concerns related to the swelling. Although sclerotherapy is a popular option for managing cervicofacial malformations, it poses various risks, including severe skin complications, peripheral nerve damage, and life-threatening events like cardiac arrest and pulmonary emboli [5]. This case report describes a unique instance of a cervicofacial VM treated successfully with a single-stage surgical excision, resulting in the complete resolution of the malformation without any complications.

## Case presentation

**Figure 1:**
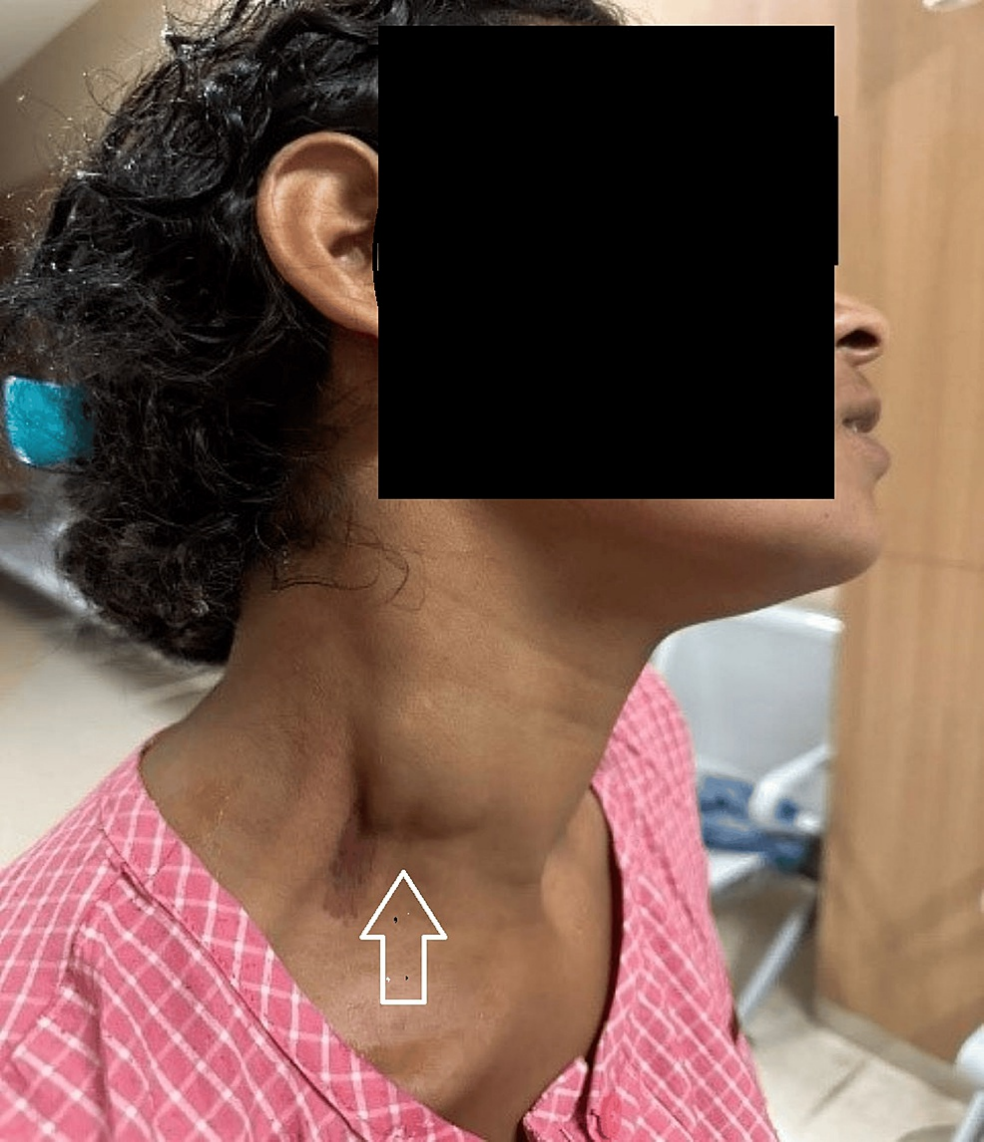
Swelling over the right side of the neck

**Figure 2:**
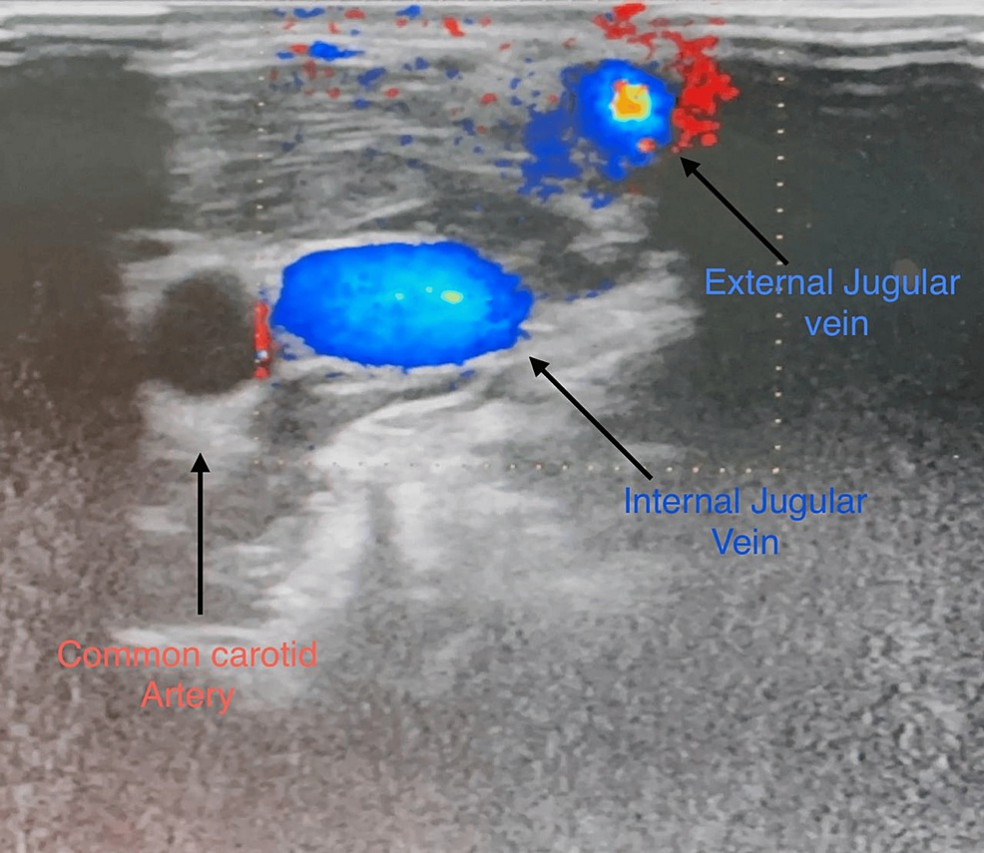
Color Doppler showing slow-flow venous malformation of the right external jugular vein

A 25-year-old female presented to the outpatient department (OPD) with a complaint of swelling on the right side of her neck for the past two years. The swelling started as a small 0.5×0.5cm mass and has gradually increased in size to approximately 4×3cm. The patient experienced occasional pain associated with the swelling, and it became more prominent when she stands after bending forward for 10-15 seconds (Video 1). Additionally, the patient reported numbness on the right side of her head and right upper limb at times. The patient's medical history revealed a traumatic incident (throttling) to the neck four years ago. Physical examination revealed a soft, non-tender, and mobile mass measuring 4×3cm in the right posterior triangle of the neck when the patient bent forward for two minutes and stood back (Figure 1). The swelling was not adhered to the underlying muscle or to the skin over it, and there was no pulsation over the swelling. Enlarged lymph nodes are not palpable in the rest of the neck. Routine laboratory blood investigations were done which were within normal limits. The patient was asked to bend forward for 2-3 minutes, then stand back, and lie down in a supine position for ultrasound with color Doppler which showed a well-defined, anechoic (echo-free) collection with multiple thin septae in the right cervical region, along the anterior aspect of the right external jugular vein, measuring approximately 28×20mm. The multiple tubular anechoic spaces within the lesion exhibited venous flow on color and spectral Doppler imaging (Figure 2).

**Video 1:** Swelling over the neck when the patient stands after bending forward for 10-15 seconds

**Figure 3:**
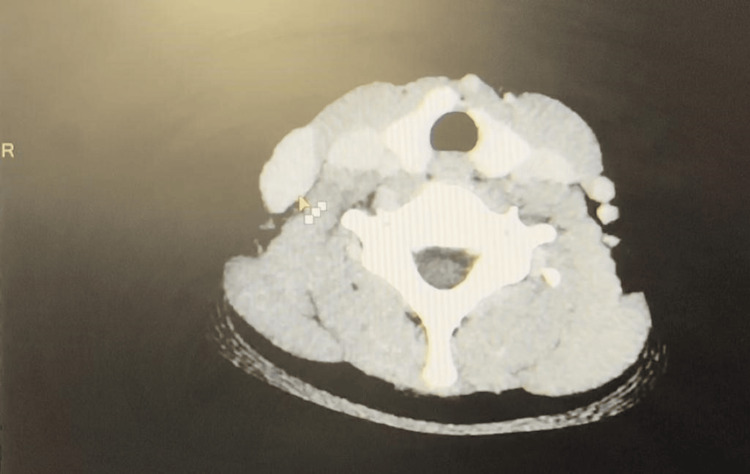
CT image showing slow-flow venous malformation of the right external jugular vein

**Figure 4:**
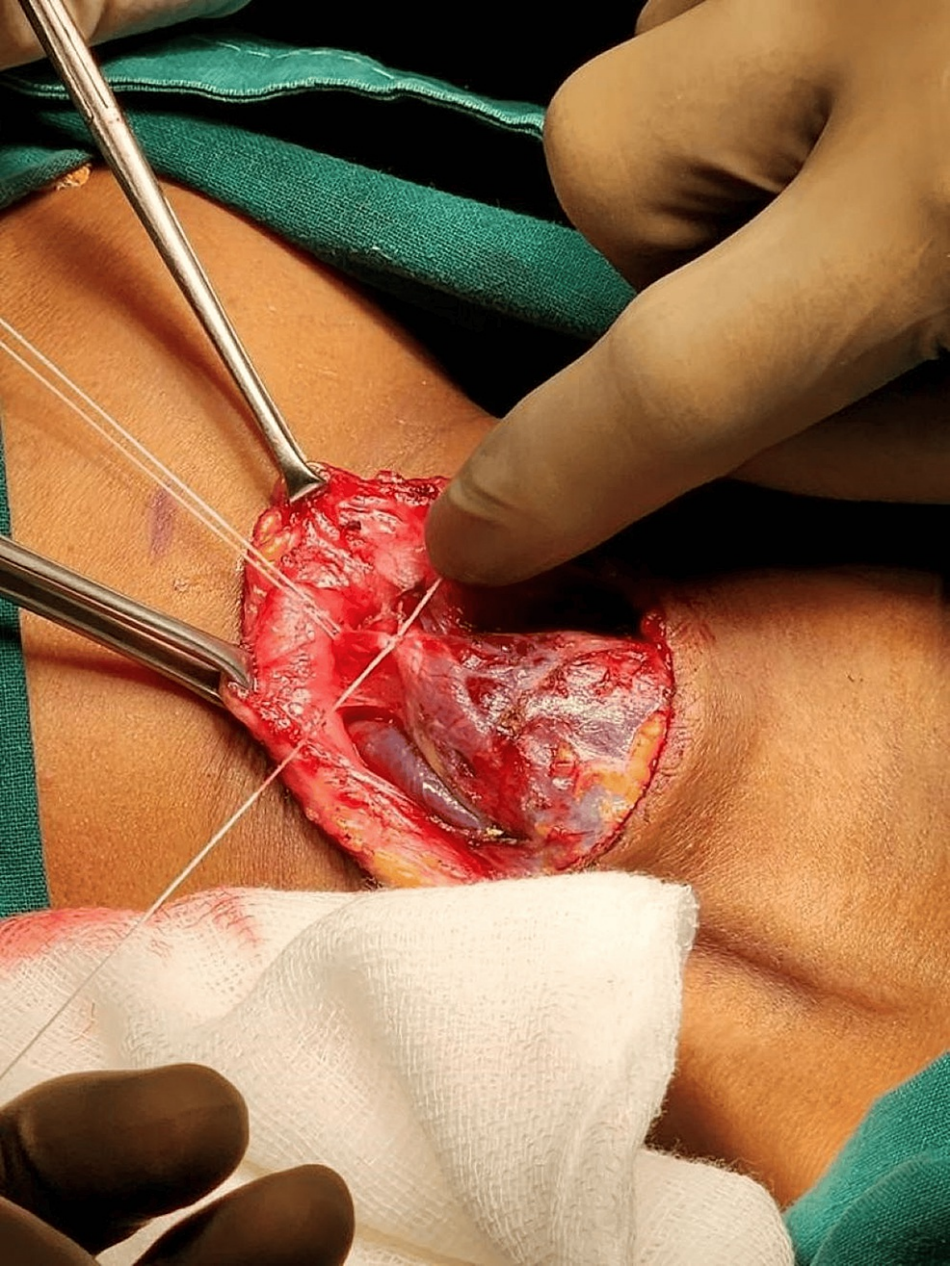
Multiple feeding vessels were ligated

The lesion was located lateral to the right sternocleidomastoid muscle and was connected to the external jugular vein. The lesion was compressible and showed no evidence of thrombosis. It increased in size when pressure was applied to the proximal part of the right external jugular vein and when the patient bent forward, confirming the diagnosis of a slow-flow VM involving the right external jugular vein. For further detailed evaluation, CT with angiography was done which revealed a well-defined oval lesion with iso- to hypodense attenuation (40-45 HUI) in the subcutaneous plane of the right lower cervical region, along the anterior aspect of the right external jugular vein (Figure 3). The lesion measured approximately 35×28×20mm, was inseparable from the external jugular vein on the posterior aspect, and showed contrast intravasation from the vein during the early venous phase. Subsequent delayed scans showed gradual contrast filling and homogeneous enhancement of the lesion after six minutes. The lesion was causing indentation and displacement of adjacent structures, including the sternocleidomastoid muscle and tributaries of the right external jugular vein, leading to a contour bulge on the skin surface. There was no evidence of calcification. Based on the ultrasonography and CT angiography findings, the diagnosis was confirmed as a slow-flow VM intimately connected with the right external jugular vein. During surgery, the VM of size measuring approximately 4x3cm arising from the external jugular vein was identified. Adhesions were released over the external jugular vein, two venous tributaries ligated superior to the VM and one tributary ligated inferior to the VM. Multiple feeding vessels were found and ligated (Figure 4), and the lesion was completely excised while preserving the normal external jugular vein.

**Figure 5:**
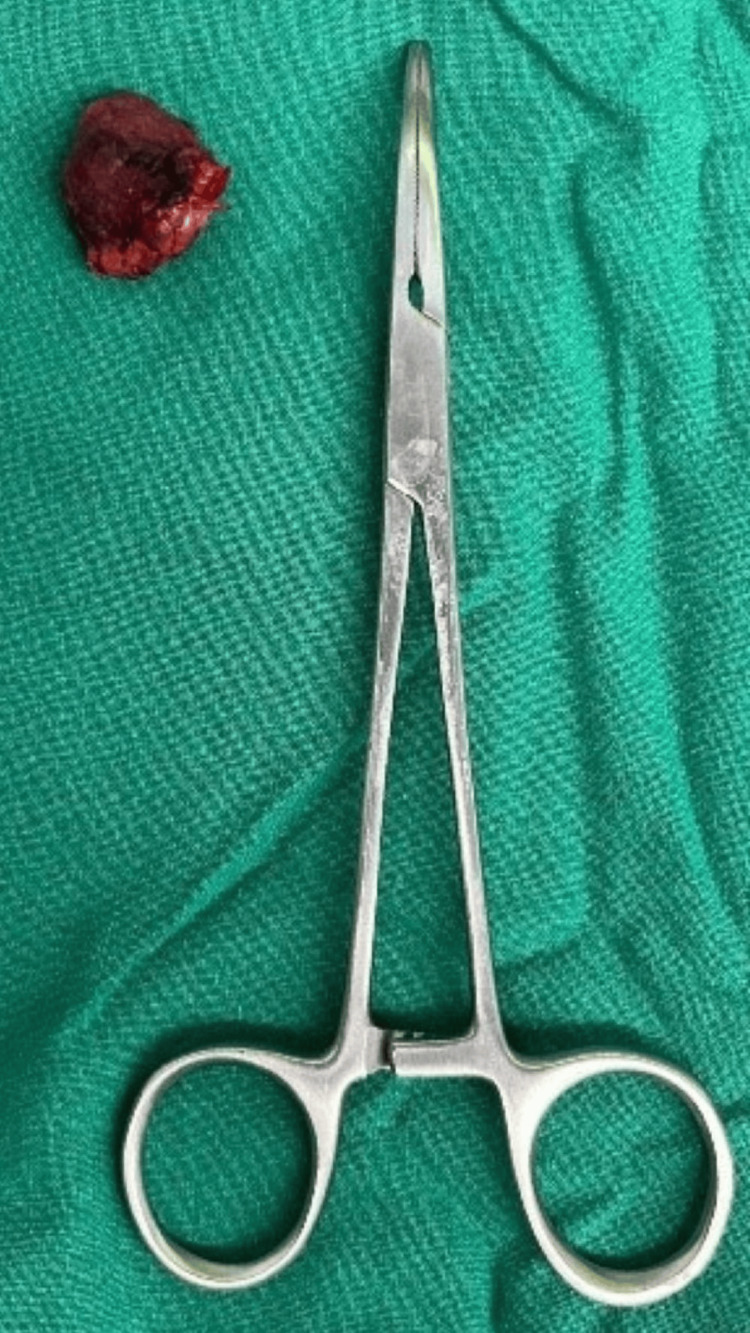
Excised lesion

Macroscopically, the excised lesion was well-circumscribed and brown in color (Figure 5). Microscopic examination of the lesion revealed large, dilated vessels lined by flattened endothelium. There was no evidence of cytologic atypia or mitosis. Focal thrombi with hyalinization were observed, and the vascular spaces were separated by fibrous septa containing small vessels. No intervening parenchyma was present. The surrounding tissue showed features of fibrofatty and fibromuscular tissue. In conclusion, the patient's symptoms and imaging findings were indicative of a slow-flow VM involving the right external jugular vein, and a successful surgical excision was performed to remove the lesion. 

## Discussion

Vascular lesions are classified into two types based on distinct clinical characteristics, that is, hemangiomas and vascular malformations. Hemangiomas present usually as a small red mark that is present at birth in almost 40% of the cases [6]. They show rapid neonatal growth which is characterized by endothelial cellular hyperplasia and proliferation in the proliferative phase. Vascular malformations are mostly identified at birth in 90% of the cases. They grow in proportion with age and do not regress. On histological examination, they are characterized by "mature" endothelium which is not hypercellular and which shows normal endothelial cell cycle. They show multiple combinations of arteries, veins, capillaries, and lymphatics, with or without fistula formation. These malformations are mostly VMs. Analysis by cell orientation has gained a large range of acceptance due to its application in diagnosis that helps in the plan of treatment [7,8]. VMs have an incidence of 1-2 per 10000 births with a 1% prevalence that is presented at birth and that has grown in proportion with age leading to various types of clinical presentations [4,9]. Slow-flow VMs in association with the external jugular vein are unusual [10]. Below 10 presentations have been noted [3]. VMs are associated with the external jugular vein through a single vein or by multiple venous connections that are mostly of large size in caliber. Histological anomalies of the pericyte component of the smooth muscle within the channel walls of VMs are to be considered as a likely reason for many VMs. Jackson and his co-workers had identified the requirement for further additions in the classification of Mulliken and Glowacki by answering the (therapeutic) questions (1) "What to do?" and (2) "When to do it?". On many occasions, overenthusiasm and incomplete treatment can lead to more worsening of the features. Morbidity after treating the patient can cause more distress for the patients in comparison to their status before treating them. Therapy is the indication in patients with malformations causing pain and patients who have functionally and aesthetically impaired malformations. Pain is manifested usually because of the engorgement of veins and the stretching of tissues locally as in our case [10]. Over the last decade, sclerotherapy has gained more popularity in treating cervicofacial VMs. The concentration of the sclerosant and the period of dwell, when there are many wide communication channels between the VM and the vein associated with it, are important predictors of successful sclerotherapy [3,11]. Due to the high risk of necrosis of the skin, unsuccessful rates of sclerotherapy, and external jugular vein proximity, surgical excision is executed in our presentation. Surgical excision and skin closure with the subcuticular method of suturing in our patient gave excellent results cosmetically. 

## Conclusions

In the vascular malformations that arise from the external jugular vein, the vein is usually normal, and it can be spared during the excision. Surgical excision of these lesions should be contemplated as the first line of treatment for VMs. The advantages of surgery are the least morbidity, least downtime, and low rates of recurrence rates. 

## References

[1] Mulliken JB, Glowacki J (1982). Hemangiomas and vascular malformations in infants and children: a classification based on endothelial characteristics. Plast Reconstr Surg.

[2] Jung HL (2021). Update on infantile hemangioma. Clin Exp Pediatr.

[3] Ahuja AT, Yuen HY, Wong KT (2004). External jugular vein vascular malformation: sonographic and MR imaging appearances. AJNR Am J Neuroradiol.

[4] Legiehn GM, Heran MK (2008). Venous malformations: classification, development, diagnosis, and interventional radiologic management. Radiol Clin North Am.

[5] Cho BC, Kim JB, Lee JW (2016). Cervicofacial lymphatic malformations: a retrospective review of 40 cases. Arch Plast Surg.

[6] Drolet BA, Esterly NB, Frieden IJ (1999). Hemangiomas in children. N Engl J Med.

[7] Meyers MA (1967). Hemangioma of the external jugular vein. Radiology.

[8] Sarteschi LM, Bonanomi G, Mosca F, Ferrari M (1999). External jugular vein hemangioma occurring as a lateral neck mass. J Ultrasound Med.

[9] Lee IH, Kim KH, Jeon P (2009). Ethanol sclerotherapy for the management of craniofacial venous malformations: the interim results. Korean J Radiol.

[10] Olivares JL, Rodríguez G, Fernández JA, de Gregorio MA (2001). Jugular venous malformation in an 8-year-old boy: treatment with endovascular sclerotherapy. Eur J Pediatr.

[11] Odeyinde SO, Kangesu L, Badran M (2013). Sclerotherapy for vascular malformations: complications and a review of techniques to avoid them. J Plast Reconstr Aesthet Surg.

